# Sex with animals among men attended in referral centers for sexually transmitted infections in northeast Brazil: prevalence, associated factors and behavioral aspects

**DOI:** 10.1590/S1677-5538.IBJU.2022.0395

**Published:** 2023-02-10

**Authors:** Lucineide Santos Silva Viana, Vinicius Fernando Calsavara, Fernanda Monteiro Orellana, Luciana Paula Fernandes Dutra, Venâncio de Sant’Ana Tavares, Stênio de Cássio Zequi

**Affiliations:** 1 Departamento de Enfermagem Universidade Federal do Vale do São Francisco Petrolina PE Brasil Departamento de Enfermagem, Universidade Federal do Vale do São Francisco - UNIVASF, Petrolina, PE, Brasil;; 2 Department of Biostatistics and Bioinformatics Samuel Oschin Cancer Center Los Angeles CA USA Department of Biostatistics and Bioinformatics, Samuel Oschin Cancer Center, Cedars-Sinai, Los Angeles, CA, USA;; 3 Departamento de Urologia Universidade de São Paulo São Paulo SP Brasil Departamento de Urologia, Universidade de São Paulo - USP, São Paulo, SP, Brasil;; 4 Departamento de Urologia AC Camargo Cancer Center São Paulo SP Brasil Departamento de Urologia, AC Camargo Cancer Center, São Paulo, SP, Brasil;; 5 Instituto Nacional de Ciência e Tecnologia em Oncogenômica e Inovação Terapêutica AC Camargo Cancer Center São Paulo Brasil Instituto Nacional de Ciência e Tecnologia em Oncogenômica e Inovação Terapêutica, INCIT/INOTE - AC Camargo Cancer Center, São Paulo, Brasil;; 6 Escola Paulista de Medicina UNIFESP São Paulo SP Brasil Pós-Graduação Stricto Sensu em Urologia, Escola Paulista de Medicina - EPM, Universidade Federal de São Paulo - UNIFESP, São Paulo, SP, Brasil

**Keywords:** Sexually Transmitted Diseases, Genital Diseases, Male, Sexual Behavior

## Abstract

**Purpose:**

Our objective was to investigate the prevalence of SWA, associated factors, relationship with STIs, and behavioral aspects in men attended at Referral Centers for STIs and acquired immunodeficiency syndrome (AIDS)/CR-STI/AIDS in northeast Brazil.

**Materials and Methods:**

In this cross-sectional study, a questionnaire with sociodemographic, clinical, sexual and SWA practices information was applied to 400 men attended at two CR-STI/AIDS in Northeast Brazil on the years of 2018 and 2019. Clinical and laboratory diagnoses of STIs were confirmed in medical records. Logistic regression models were performed to identify the independent predictors for SWA.

**Results:**

The prevalence of SWA over total samples was 15.00%. Of the participants, 239 (59.75%) of the participants were diagnosed with STIs, and of these 37 (15.48%) reported SWA. Most men practiced SWA in adolescence, being the last episode more than 20 years ago, usually with asinine and mules, in vaginal route and without a condom. SWA practitioners have higher percentages of occurrence of some viral STIs. SWA was associated with increasing age, history of residence in a rural area with remained over 12 years, married or widowed/separated, heterosexuals, with less than 7 years of study, Catholics, with hepatitis B, former user of alcoholic beverages and smokers, with a history of STI and intercourse with sex workers.

**Conclusion:**

SWA practices increase STIs vulnerability. The association between hepatitis B and SWA highlights the importance of educational campaigns and conclusive studies on the topic.

## INTRODUCTION

The term “Sex With Animals” (SWA) has been used to portray human sexual behavior, without reinforcing the moral stereotypes that permeate the term bestiality or associating it with a medical diagnosis, such as zoophilia or zoophilic disorder ( 1 ). In these situations, the paraphilic disorder causes suffering or harm to the individual, in addition to the possibility of harming himself or others for his satisfaction ( 2 ).

Researchers suggest that this sexual interest may be triggered by hypersexuality associated with dementia ( 3 ) or secondary to drugs used to treat Parkinson’s Disease ( 4 ). Behavioral factors such as autism spectrum disorder are also mentioned ( 5 ) and some psychiatric disorders ( 6 , 7 ), and identify characteristics associated with possible sexual orientation ( 8 ). Although the SWA practice can result in health damage such as arthritis ( 9 ), herpes B ( 10 ), anogenital traumas ( 11 ), and penile cancer ( 1 ), few studies relate this behavior to Sexually Transmitted Infections (STIs).

In sexual relations between humans and animals, injuries resulting from the disproportionate size of the external genital organs ( 11 ), damage genital tissues caused by bites and scratches, and secondary traumas while attempting to disengage from the animal with penile dilation on penetration ( 12 ) can increase vulnerability to infections. The risk is even more remarkable when humans assume the receptive position in anal sex because of the fragility of the human rectal mucosa and the absence of a protective immune barrier such as the cervicovaginal secretions ( 13 ). STI of an animal pathogen for people was proven in Budapest, with *Kurthia gibsonii* as an etiologic agent, a bacterium present in swine feces, isolated in the urethra and glans of an adult individual ( 14 ).

Since the Kinsey studies ( 15 ) when an 8% prevalence of zoophilia was established among American men (usually from rural areas), there has been an attempt to estimate the occurrence of this sexual practice in other population groups. In Brazil, SWA was reported by 3.2% of the adult population ( 16 ). The Northeast region has one of the highest rates, 4.5%, which may be related to the more significant extension of rural areas. Research that included Northeastern men with SWA practice identified a history of STI, without clinical or laboratory proof, greater than 50% of the sample ( 1 , 17 ). We hypothesize that men with a history or practice of SWA have more records of STI occurrences than those without SWA. Given this scenario, the present study analyzed the prevalence of SWA, its associated factors, its relationship with STIs, and behavioral aspects in men attended at Refer Centers for STIs and acquired immunodeficiency syndrome (AIDS) / CR-STI/AIDS in northeastern Brazil.

In Brazil, there is no specific legislation that prohibits sexual acts between humans and animals. However, abusive situations that promote mistreatment, injuries or mutilations are considered environmental crimes and can be penalized with a minimum detention of 3 months which can reach 5 years - in the case of dogs and cats. In these cases, the death of the animal can increase the penalty by up to 1 third ( 18 ). Legislators analyze the approval of a bill that typifies and criminalizes zoophilia, regardless of physical injuries. In this case, erotic and/or sexual acts can be penalized with imprisonment of up to five years ( 19 ). Those who mediate or publicly expose sexual acts between humans and animals can also be penalized ( 20 ). In most American states, several countries in Europe, Iran and other Islamic countries SWA are considered a crime ( 12 ).

## MATERIALS AND METHODS

This study was approved by the Research Ethics Committee of Federal University of Vale do São Francisco (UNIVASF), number 2.133.407. We conducted a cross-sectional study with men treated at two Referral Centers for Sexually Transmitted Infections and AIDS (CR-STI/AIDS) located in Juazeiro (Bahia) and Petrolina (Pernambuco), both in Northeast Brazil. The service offers STI prevention actions, diagnosis, treatment, and monitoring of these conditions, with rapid testing for HIV, syphilis, Hepatitis B, and C performed on all new entrants. Data collection took place over 24 months (2018 and 2019) and included men over 18 years of age, regardless of serological or syndromic conditions for HIV or other STIs. The study excluded people with mental or intellectual disabilities because they pose risks to the veracity of the information or difficulties in expressing themselves.

Considering the scarcity of previous studies to estimate the population of men with SWA practice attended in CR-STI/AIDS, one researcher collected data with as many participants as possible in the pre-established two-year period. We approached 542 men in waiting rooms recruited at random and taken to a restricted location. The non-probabilistic sample comprised 400 individuals who accepted the invitation and answered a structured questionnaire built by the researchers with questions about sociodemographic data, alcoholism, and smoking, sexuality, STI/AIDS, sex with animals, the current condition of the anogenital region, and results of rapid tests for HIV, syphilis, and hepatitis B and C. All subjects provided written informed consent.

### Variables

The sociodemographic variables selected were age, residence history in rural areas, time lived in rural areas, race, marital status, schooling, and religion. We also analyzed smoking, use of alcoholic beverages, duration of alcohol consumption/year, use of illicit drugs in the last year, age of first sexual intercourse, sexual orientation, sexual relations with sex workers, and occurrence of current anogenital complaints according to a medical record.

We investigated the time that the individual “has been living with HIV” (less than 1 year, from 1 to 3 years, from 4 to 6 years, from 7 to 9 years, from 10 to 19 years, and ≥ 20 years) and the occurrence of “hepatitis B + HIV + hepatitis C” coinfection.

In “STI patients,” the following cases were validated as sexually transmitted infections with clinical or laboratory diagnostic evidence registered in the medical record: genital herpes, chancroid, anogenital warts (caused by the human papilloma virus-HPV), and other STIs. In this last category, only candidiasis was included when the possibility of an endogenous condition was ruled out. Urethral Discharge Syndrome (UDS) was inserted to cover urethritis, since in CR-STI/AIDS these cases are diagnosed, treated, registered in medical records, and notified as UDS. We considered syphilis situations that followed the criteria established by the Brazilian Ministry of Health (MH) for asymptomatic and symptomatic individuals ( 21 ). HIV infection cases ( 22 ), Human T-lymphotropic virus- HTLV, Hepatitis B and C ( 21 ) followed only laboratory diagnosis recommended by the MH.

SWA issues involved the date of the last episode; the age of onset and termination of these sexual practices; species and sex of the animal used; frequency of SWA relationships (weekly, monthly, yearly, and only once in a lifetime intervals); possibility of variation of the animals at each sexual intercourse; presence or absence of human companions during SWA; a sexual position assumed by the individual (insertive, receptive or both); type of sexual practice performed (vaginal, anal, oral, masturbation, others); condom use (not always, more than half the time, less than half the time); internet access to search for SWA content (yes, no); type of virtual content accessed (pornographic films, images, social networks) and SWA versus HIV infection (SWA only before HIV diagnosis, SWA only after HIV diagnosis, SWA before and after HIV diagnosis).

### Statistical Analysis

The patients’ characteristics were expressed as absolute and relative frequencies for qualitative and mean, median, range, and standard deviation for quantitative variables. The chi-squared test or Fisher’s exact test were applied to evaluate a possible association between the independent factors with the dependent (SWA) variable. The student’s t-test or Mann-Whitney U test was applied to compare the data of the quantitative variable in relation to the SWA group. Shapiro-Wilk test was applied to test the data normality.

In addition, we fitted the univariable and multivariable logistic regression models to evaluate the associations between exposure and outcome (SWA). The assumption of linearity was assessed for all continuous variables. No imputation method was used for missing data. The assessment of model significance and performance was performed through the Hosmer-Lemeshow goodness-of-fit test, receiver operating characteristics (ROC) curve, and c-statistic, representing the area under the ROC curve (AUC). The significance level of the tests was fixed at 0.05 (two-sided). All analyses were performed using the R software 4.0 version (R Foundation for Statistical Computing, Vienna, Austria).

## RESULTS

The prevalence of SWA practice among the participants was 15% (n = 60), 95%CI= [11.65% - 18.88%]. These men were older (50.07, standard deviation-SD: 13.65) than those who denied SWA (35.41, SD: 13.92, *p* ˂ 0.0001), usually married or living in a stable union (58.33%, *p* = 0.012), catholic (63.33%, *p* = 0.031), self-defined blacks (black or brown, 85.00%, *p* = 0.564), heterosexual (91.67%, *p* = 0.023), less schooling (70% with 0 to 7 years of study, *p* ˂ 0.0001) with 10 (16.7%) illiterate SWA men (versus 15, 4.4% among non-SWA men). They lived in a rural area during childhood or adolescence (86.67%, *p* ˂ 0.0001), where they generally stayed for more than 12 years (78.85%, *p* = 0.002). They also sought more sex with sex workers (43.33%) than non-SWA men (14.71%, p < 0.0001) Table-1 .

**Table 1:** Sociodemographic, clinical, and sexual characteristics of men with SWA practice and without SWA practice attended at the CR-IST/AIDS of Juazeiro-BA and Petrolina-PE in the years 2018 and 2019, Brazil.

Variable	Category	Total sample n=400		Men with SWA practice n=60		Men without SWA practice n=340		p
**Age (years)**	Mean (SD)	37.61	(14.8)	50.07	(13.65)	35.41	(13.92)	< 0.0001
	Median (Range)	34	(18-83)	51.50	(21-78)	32.00	(18-83)	
**Marital status**	Not married	196	(49.00%)	19	(31.67%)	177	(52.06%)	0.012
	Married/stable relationship	179	(44.75%)	35	(58.33%)	144	(42.35%)	
	Widowed/Separated/ Divorced	25	(6.25%)	6	(10.00%)	19	(5.59%)	
**Religion**	Without religion	114	(28.50%)	10	(16.67%)	104	(30.59%)	0.031
	Catholic	194	(48.50%)	38	(63.33%)	156	(45.88%)	
	Others^a^	92	(23.00%)	12	(20.00%)	80	(23.53%)	
**Race**	Blacks^b^	326	(81.50%)	51	(85.00%)	275	(80.88%)	0.564
	Others^c^	74	(18.50%)	9	(15.00%)	65	(19.12%)	
**Schooling**	From 0 to 7 years of study	153	(38.25%)	42	(70.00%)	111	(32.65%)	< 0.0001
	Over 8 years of study	247	(61.75%)	18	(30.00%)	229	(67.35%)	
**Living in a rural area**	No	201	(50.25%)	8	(13.33%)	193	(56.76%)	< 0.0001
	Yes	199	(49.75%)	52	(86.67%)	147	(43.24%)	
**Time lived in rural area**	Less than 3 years	36	(18.09%)	4	(7.69%)	32	(21.77%)	0.002
	From 4 to 11 years	48	(24.12%)	7	(13.46%)	41	(27.89%)	
	More than 12 years	115	(57.79%)	41	(78.85%)	74	(50.34%)	
**Use of alcoholic beverages**	No	54	(13.50%)	5	(8.33%)	49	(14.41%)	0.012
	Social drinker	180	(45.00%)	21	(35.00%)	159	(46.76%)	
	Ex drinker	85	(21.25%)	22	(36.67%)	63	(18.53%)	
	Current drinker	81	(20.25%)	12	(20.00%)	69	(20.30%)	
**Duration of alcohol consumption/ years**	Mean (SD)	15.02	(12.14)	22.82	(13.80)	13.01	(10.85)	< 0.0001
	Median (Min-Max)	10	(1-55)	22.50	(1-55)	10	(1-50)	
**Smoking**	No smokers	267	(66.75%)	29	(48.33%)	238	(70.00%)	< 0.0001
	Smokers	76	(19.00%)	13	(21.67%)	63	(18.53%)	
	Ex smokers	57	(14.25%)	18	(30.00%)	39	(11.47%)	
**Use of illicit drugs in the last year**	No	314	(78.50%)	49	(81.67%)	265	(77.94%)	0.633
	Yes	86	(21.50%)	11	(18.33%)	75	(22.06%)	
	Cannabis	65	(16.25%)	6	(10.00%)	59	(17.35%)	0.217
	Cocaine	43	(10.75%)	8	(13.33%)	35	(10.29%)	0.635
	Crack	9	(2.25%)	3	(5.00%)	6	(1.76%)	0.139
	Volatile solventes (shoe glue)	1	(0.25%)	0	(0.00%)	1	(0.29%)	0.999
	Injecting drugs	5	(1.25%)	0	(0.00%)	5	(1.47%)	0.999
**Current anogenital condition**	No changes or complaints	265	(66.25%)	43	(71.67%)	222	(65.29%)	0.415
	Yes	135	(33.75%)	17	(28.33%)	118	(34.71%)	
**Sexual orientation**	Heterosexual	320	(80.00%)	55	(91.67%)	265	(77.94%)	0.023
	No heterosexual	80	(20.00%)	5	(8.33%)	75	(22.06%)	
**Sex with sex workers**	No	324	(81.00%)	34	(56.67%)	290	(85.29%)	< 0.0001
	Yes	76	(19.00%)	26	(43.33%)	50	(14.71%)	
**Age of 1st sexual intercourse**	Mean (SD)	15.10	(2.76)	15.18	(3.06)	15.08	(2.71)	0.795
	Median (Min-Max)	15	(5-23)	16	(8-22)	15	(5-23)	

^a^ includes blacks and browns

^b^ includes white, yellow and indigenous

^c^ includes Evangelical, Protestant, Spiritist, Candomblé and Umbanda

Participants with SWA reports had higher percentages for “ex drinker” (36.67%), while the majority without a history of SWA identified themselves as “social drinkers” (46.76% *, p* = 0.012). The average length of consumption (in years) was also longer among the group with SWA practice (22.82 years - SD: 13.8, versus 13.01 years - SD: 10.8, p < 0.0001). Most respondents denied using nicotine cigarettes (66.75%, *p* < 0.0001). We did not identify any statistically significant differences between men with and without a SWA report for the categories related to the use of illicit drugs. At the time of the interview, only 33.75% of the interviewees reported anogenital complaints ( *p* = 0.415), usually urethral discharge, warts, and vesicles Table-1 .

For most respondents, the first sexual intercourse with humans occurred on average at 15.10 years of age (SD: 2.76, p = 0.795), while SWA practices started at around 12.37 (SD: 3.78) years and ended at 16.43 (SD: 7.86) years ( Table-1 ), having had the last SWA intercourse more than 20 years ago (n = 47, 78.33%). There were no records for the last SWA in the year leading up to the survey. Participants mentioned multiple responses for the animals most used during SWA. There was a predominance of females (n = 56, 93.33%), asinine and mule species (n = 46, 76.66%), goats (n = 27, 45,00%) and chickens (n = 20, 33.33%) Table-2 .

**Table 2:** Characterization of SWA practice among men treated at CR-IST/AIDS in Juazeiro-BA and Petrolina-PE (Northeastern Brazil) in 2018 and 2019.

Variable	Category	n	%
**SWA**	Yes	60	15.00
**Last SWA**	1 to 4 years ago	3	5.00
	5 to 9 years ago	0	0.0
	10 to 19 years ago	10	16.67
	More than 20 years	47	78.33
**Animal**	Asinines and mules	46	76.66
	Goats	27	45.00
	Gallinaceous	20	33.33
	Calf	15	25.00
	Horses (Horse / mare)	13	21.66
	Sheep	11	18.33
	Adult Cattle	7	11.66
	Swine	4	6.66
	Canine	1	1.66
	Feline	1	1.66
	Duck / mallard / goose	1	1.66
**Animal sex**	Only Females	56	93.33
	Only males	1	1.67
	Females and males	3	5.00
**Exposure time (years)**	Mean initial age (DP)	12.37 (3.78)	
	Median (Min-Max)	12 (7-31)	
	Mean final age (DP)	16.43 (7.86)	
	Median (Min-Max)	15 (7-60)	
**Frequency**	Once to 3 times a week	19	31.67
	Once to twice times a month	11	18.33
	Once every 2 months	6	10.00
	2 to 4 times a year	7	11.67
	Anual	6	10.00
	Once in a lifetime	11	18.33
**Variation of animals with each coitus**	Always with the same animal	28	46.67
	Different animals in coitus	32	53.33
**Presence of human companionship during SWA**	Generally individual	35	58.33
	Generally, in a group	22	36.67
	Both	3	5.00
**Position during SWA**	Insertive	59	98.33
	Receptive	0	0
	Both	1	1.67
**Type of relationship with the animal**	Vaginal	58	96.67
	Oral	0	0.0
	Anal	5	8.33
	Masturbation	3	5.00
**Condom use during SWA**	No	57	94.99
	Always	1	1.67
	More than half the time	1	1.67
	Less than half the time	1	1.67
**Uses internet to search for SWA content**	No	43	71.67
	Yes	17	28.33
**Type of content searched for on the internet related to SWA**	Porn movies	16	94.12
	Imagens	9	52.94
	Social networks	0	0.0
**SWA X HIV infection**	Not applicable	45	75.00
	SWA only prior to HIV diagnosis	14	23.33
	SWA only after HIV diagnosis	0	0.0
	SWA before and after HIV diagnosis	1	1.67

The SWA frequency was generally 1 to 3 times a week (n = 19, 31.67%). Most sexual intercourse occurred with different animals at each episode (n = 32, 53.33%), usually the men were alone with the animal (n = 35, 58.33%), in an insertive position (n = 59, 98.33%). Among the mentioned mammals, the main sexual intercourse was vaginal (n = 58, 96.67%), without using condoms (n = 57, 94.99%). The minority of participants used internet to access content about SWA (n = 17, 28.33%) such as pornographic films (n = 16, 94.12%) and images (n: 9, 52.94%). Everyone denied access to social networks on SWA. Among the 15 HIV-infected SWA men, 14 of them stated that sex with animals occurred only before diagnosis and only 1 declared SWA before and after.

We identified 179 (46.49%, *p* = 1.000) men in the sample with one or more STIs, with clinical or laboratory evidence, in the 12 months preceding the research. This variable totaled a sample of 385 participants because 15 of them had no documentary evidence of STI and were excluded. Among the participants, 239 (59.75%, *p* = 0.853) had STIs, and of these, 37 (61.67%) reported a history of SWA. Only the hepatitis B category had a statistically significant difference between the groups of SWA and non-SWA men ( *p*: 0.048) Table-3 .

**Table 3:** Characteristics related to STIs among the groups of men who stated and denied SWA practice attended at the CR-IST/AIDS in Juazeiro-BA and Petrolina-PE (Northeastern Brazil) in 2018 and 2019.

Variable	Category	n	(%)	Men with SWA practice	(%)	Men without SWA practice	(%)	P
**STI in the last year**	No	206	(53.51)	31	(53.45)	175	(53.52)	0.999
	Yes	179	(46.49)	27	(46.55)	152	(46.48)	
**STI older than 12 months**	No	206	(52.42)	19	(31.67)	187	(56.16)	0.001
	Yes	187	(47.58)	41	(68.33)	146	(43.84)	
**STI carriers**	Yes	239	(59.75)	37	(61.67)	202	(59.41)	0.853
	No	161	(40.25)	23	(38.33)	138	(40.59)	
	UDS	47	(11.75)	4	(6.67)	43	(12.65)	0.267
	Syphilis	53	(13.25)	5	(8.33)	48	(14.12)	0.312
	Warts /HPV	18	(4.50)	2	(3.33)	16	(4.71)	0.999
	HIV	89	(22.25)	15	(25.00)	74	(21.76)	0.699
	Hepatitis B	10	(2.50)	4	(6.67)	6	(1.76)	0.048
	Chancroid	2	(0.50)	0	(0.00)	2	(0.59)	0.999
	Genital herpes	14	(3.50)	4	(6.67)	10	(2.94)	0.242
	Hepatitis C	36	(9.00)	9	(15.00)	27	(7.94)	0.129
	HTLV	1	(0.25)	0	(0.00)	1	(0.29)	0.999
	Others (candidiasis)	5	(1.25)	1	(1.67)	4	(1.18)	0.558
**Living with HIV**	No	311	(77.75)	45	(75.00)	266	(78.24)	0.247
	Less than 1 year ago	24	(6.00)	3	(5.00)	21	(6.18)	
	1 to 3 years	26	(6.50)	2	(3.33)	24	(7.05)	
	4 to 6 years	10	(2.50)	3	(5.00)	7	(2.06)	
	7 to 9 years	4	(1.00)	0	(0.00)	4	(1.18)	
	10 to 19 years	19	(4.75)	6	(10.00)	13	(3.82)	
	over 20 years	6	(1.50)	1	(1.67)	5	(1.47)	
**HIV+HBV+HCV Co-infection**	No	397	(99.25)	58	(96.67)	339	(99.71)	0.060
**HIV+HBV+HCV Co-infection**	Yes	3	(0.75)	2	(3.33)	1	(0.29)	

Among individuals living with HIV (n = 89, 22.25 %%), we observed a trend that those with a history of SWA accumulated higher percentages in periods of seropositivity greater than ten years (11.67%) while in the others diagnoses prevailed in the last six years (13.33%, *p* = 0.247). HIV + HBV (hepatitis B virus) + HCV (hepatitis C virus) coinfection cases were not significantly higher among SWA men (3.33% versus 0.29%, *p* = 0.060) than among those who denied such behavior.

The univariate analysis revealed that the greater probability of developing SWA practices is associated with increasing age; rural area residence history, mainly with an over-12 years permanence; being married or widowed/separated; heterosexual; with less than seven years of study; catholic; ex-alcoholic drinker and cigarette; having a history of sexual relations with sex workers and STIs throughout life and having hepatitis B. The study excluded from this simple logistic regression model the variable “duration of alcohol consumption” because it considered another one related to the theme of alcoholism ( Table-4 ).

**Table 4:** Univariable logistic regression model for the primary outcome (SWA practices).

Variable	Category	OR	95% CI		p value

			Lower	Upper	
**Age (years)**	unit increment	1.066	1.046	1.088	< 0.0001
**Rural area inhabitant**	Yes	8.534	3.933	18.518	< 0.0001
**Time lived in a rural area**	≤3 years	Ref			
	4 to 11 years	1.366	0.368	5.075	0.642
	≥12 years	4.432	1.465	13.413	0.008
**Marital status**	Single	Ref			
	Married/stable relationship	2.264	1.242	4.127	0.008
	Widower/separated	2.942	1.048	8.262	0.041
**Schooling**	Over 8 years	Ref			
	From 0 to 7 years of study	4.814	2.65	8.744	< 0.0001
**Religion**	Non-religious	Ref			
	Catholic	2.533	1.209	5.307	0.014
**Consumption of alcoholic beverages**	No use	Ref			
	Ex drinkers	3.422	1.209	9.685	0.020
**Smoking**	Non-smokers	Ref			
	Ex-smokers	3.788	1.922	7.466	< 0.0001
**Sexual orientation**	Heterosexual	3.113	1.203	8.056	0.019
**Sex with sex workers**	Yes	4.435	2.453	8.02	< 0.0001
**STI older than 12 months**	Yes	2.637	1.47	4.731	0.001
**Having hepatitis B**	Yes	3.976	1.088	14.538	0.037

The multivariable logistic regression model indicated that men who were more likely to have sex with animals were older (OR = 1.061, 95% CI = 1.039 - 1.084; p < 0.0001), resided in a rural area (OR = 7.163, 95% CI = 3.174 - 16.164; p < 0.0001) and had sex with sex workers in the last year (OR = 2.861, 95% CI = 1.463 - 5.594; p = 0.002). Discrimination analysis of the model showed a c-statistic of 85.4 (95% CI = 81-90) and the calibration showed a very good matching (Hosmer-Lemeshow test: χ^2^ = 7,493; df=8; p value = 0.485).

## DISCUSSION

The SWA prevalence in the surveyed men (15%) exceeds the percentage found among Americans (8%) of the Kinsey sample ( 15 ), however, it was lower than the Brazilian series ( 1 , 17 ). We also obtained a higher prevalence percentage in men with STIs with a history of SWA (15.48%) than a survey in Pakistan that identified this sexual behavior in 0.5% of a sample of 465 men with sexually transmitted infections ( 23 ).

The sociodemographic variables associated with SWA are similar to literature or to medical evidence reported ( 1 , 17 ): men with low schooling, married, and who lived in rural areas, especially in childhood. These characteristics differ from those observed in individuals with SWA practice through studies that used virtual methodologies ( 24 ): adults, singles, with more than eight years of study, living in urban centers. We do not know, however, whether there is a transition in this profile due to the rural exodus and sexual freedom seen in large cities, or whether this is the reflection of digital approaches, easing access to these populations and, at the same time, hampering the less educated, of rural origins and who do not access virtual digital groups, as verified in our investigation.

The behavioral aspects of the SWA practice demonstrated similarities with findings from other studies, such as limited exposure to adolescence ( 1 , 15 , 25 ), a predilection for farm animals ( 1 , 25 ), and weekly frequency ( 1 ). However, the interest in diversifying the animals at each intercourse and prioritizing performing them at the individual level differed from the previous findings ( 1 ). Considering that the region we investigated has numerous herds of goats and sheep, the variation of animals at each coitus may reflect the supply of available specimens. The interest in practicing SWA in a restricted way does not exempt the possibility of later socializing it in a group or being a choice influenced by the fear of condemnation by peers, especially after marriage ( 25 ).

Although the results related SWA to alcohol consumption in the past, we did not discover whether, during sexual intercourse with animals, they were under the influence of alcohol. In this case, alcohol could be used intentionally to encourage sexual intercourse, or SWA could be a consequence of chronic alcoholism. In India, researchers believe that alcohol abuse by a teenager may have caused the death of a calf penetrated during SWA ( 26 ).

The lack of statistical significance between SWA men and non-SWA men for variables that assessed the occurrence of STIs “in the last 12 months” and “STI patients” may influence the time between the last SWA and data collection. Considering that 78.3% of those who reported SWA had their last sexual intercourse with animals more than 20 years ago, it is presumed that acute bacterial STIs conditions directly related to this sexual practice have already been cured. The present research did not investigate whether the participants remembered any anogenital discomfort after practicing SWA and how they led with possible complaints.

The statistical association between SWA and carriers of HBV adds elements to discussing the possibility of transmitting this virus between humans and animals. HBV belongs to the *Hepadnaviridae* family and includes several genera among animal species, including the *Orthohepadnavirus* genus commonly found in mammals such as non-human primates ( 27 ). There are indications of a possible variant of endemic *Hepadnavirus* in swine ( 28 ) and in chickens ( 29 ), where molecular analyzes revealed 92.2% to 97.9% similarity with human HBV. Despite this, there is no overwhelming evidence that HBV transmission can occur between human and animal species, as it is a host-specific virus ( 27 ).

Given the lack of genetic and viral findings that justify the transmission of hepatitis B between humans and animals, these results may be supported in sociodemographic, biological, and behavioral aspects. National data ( 30 ) indicates that individuals diagnosed with hepatitis B have the same predominant sex, race/color and schooling level identified in SWA men.

Exposure to genital trauma and abrasions common during SWA ( 11 ), and the consequent ease of penetration by infectious viral agents in sexual relations with humans, including HIV, may increase vulnerability to HBV and other STIs. Our prevalence of SWA men infected with HVB + HCV + HIV (3.3%,) exceeds the percentages of Africans (0.15%) ( 31 ) and injecting drug users from Iran (1.25%) ( 32 ).

In our study, behavioral aspects associated with SWA such as long-lasting consumption of alcoholic beverages, history of STI infection, sexual involvement with sex workers, and low schooling may also represent an increased risk context for STIs. In addition, most of these men have a history of residing in rural areas, where access and health care are deficient in much of the country, including low vaccination coverage for hepatitis B among adults ( 33 ).

The use of condoms during sexual activities with animals was denied by most participants who reported their last SWA during adolescence, which seems to be compatible with the age of these participants (around 50 years) and their history of living in rural areas. Many lived adolescence between the 80s and 90s when the HIV epidemic was still restricted to large urban centers and the call for condom use limited. We are pioneers in presenting data on condom use in SWA intercourse.

The men who reported regular or occasional use of condoms during sex with animals (n=3) were the only ones who reported the most recent SWA (1 to 4 years before the survey), which is consistent with the increasing stimulus to the use of condoms, for the prevention and control of HIV and other STIs. We did not investigate whether these participants used condoms frequently in sexual intercourse with humans and the reasons for using condoms in SWA acts, whether they would be to make coitus more comfortable or safer from the point of view of the transmission of human-animal diseases or animal-human.

Most HIV-seropositive men with a history of SWA reported sex with animals just before diagnosis. This decision did not privilege aspects of animal self-care or well-being because, upon receiving the diagnosis, they had ended their experiences with animals since adolescence. Their average age was 54.2 years, the last SWA occurred between 13 and 19 years of age, and most were diagnosed with HIV in the last two decades.

Our research stands out for being the first cross-sectional study on SWA and STI realized in the world, in addition to identifying a statistical association between men with SWA and hepatitis B practice. Although our sample of hepatitis B patients was small, this result can add knowledge to support conclusive studies on HBV transmission between human and animal species.

This investigation adds to the small number of studies on SWA performed through face-to-face interviews published in the last decade. We verify a growing trend of recruitment via the internet, which is vulnerable to different biases even though it is an accessible and promising strategy. We also included participants from different age groups, regardless of health status and serological status for STIs, which brings the results closer to the profile of the general population. Most of the recent research conducted in health services conducted face-to-face interviews with SWA individuals were linked to penile cancer ( 1 , 17 ).

One of the limitations of this study concerns the last SWA occurrence reporting more than 20 years ago for most participants, which may have hidden complaints associated with this sexual practice. Although this is an important topic, the cross-sectional nature of the study with behaviors that were essentially long ago limits the value of the examined and identified associations. We did not investigate the time between SWA and STI diagnosis, which could be relevant since part of these men had, on average, three years of coitus exclusively with animals before starting sex with humans. We have not investigated the use of illicit drugs throughout life, especially injectable drugs and the possible sharing of syringes and needles. We recognize the limitation of cross-sectional studies to analyze the history of STIs in this population and suggest that prospective studies be conducted for this purpose.

It is necessary to consider the inclusion of new population groups in studies that articulate SWA and STI as women, given the existence of SWA practices in large urban centers ( 24 ); indigenous, forestry, and aborigines, who traditionally live in harmony with animals and who have registered high percentages of STIs in several countries ( 34 ). Research involving young adults living in rural areas can provide important information about the current context of SWA practices in this population group and allow comparisons with studies that included young populations from well-developed urban spaces ( 24 ).

## CONCLUSIONS

Our study showed that SWA is part of the sexual repertoires of adult men treated at the investigated Reference Centers for Sexually Transmitted Infections, with a prevalence of 15.00% in the investigated sample. Among men with a clinical or laboratory diagnosis of STI, we identified the highest prevalence ever recorded in the world literature, 15.48%.

SWA is associated with some sociodemographic and behavioral aspects capable of increasing vulnerability to STIs, such as: increasing age, history of residence in rural areas and sexual involvement with sex workers. The relationship between SWA and hepatitis B may provide important support for future studies that investigate the possibility of human-animal transmission. Intersectoral actions and harm reduction strategies should be considered to ensure/promote the sexual health of those involved.

Articulated actions among professionals who assist individuals with STIs, dealing with sexual health, human sexuality, animal health, and well-being should be discussed to produce scientific knowledge on the subject, approach strategies, and assist SWA supporters. It is worth considering the ethical limits that permeate actions for harm reduction, such as condoms and intimate lubricants, the impact on animal health, and the emergence of new strains of sexually transmitted pathogens.

## ABBREVIATIONS

AIDS: Acquired Immunodeficiency Syndrome
CR-IST/AIDS: Refer Center for STI/AIDS
HBV: Hepatitis B Virus
HCV: Hepatitis C Virus
HIV: Human Immunodeficiency Virus
HTLV: Human T-lymphotropic Virus
MH: Ministry of Health
STI: Sexually Transmitted Infections
SWA: Sex With Animals
UDS: Urethral Discharge Syndrome
